# Profiles of quality of outpatient care use, associated sociodemographic and clinical characteristics, and adverse outcomes among patients with substance-related disorders

**DOI:** 10.1186/s13011-022-00511-0

**Published:** 2023-01-14

**Authors:** Marie-Josée Fleury, Zhirong Cao, Guy Grenier, Christophe Huỳnh

**Affiliations:** 1 Douglas Hospital Research Centre, 6875 LaSalle Blvd, Montreal, Quebec H4H 1R3 Canada; 2 Department of Psychiatry, McGill University, 1033 Pine Avenue West, Montreal, Quebec H3A 1A1 Canada; 3 Institut universitaire sur les dépendances, Centre intégré universitaire de santé et des services sociaux du Centre-Sud-de-l’Île-de-Montréal, 950 Louvain Est, Montreal, Quebec H2M 2E8 Canada

**Keywords:** Substance-related disorders, Outpatient service use, Quality of care profiles, Latent class analysis, Sociodemographic and clinical characteristics, Adverse outcomes

## Abstract

**Background:**

This study identified patient profiles in terms of their quality of outpatient care use, associated sociodemographic and clinical characteristics, and adverse outcomes based on frequent emergency department (ED) use, hospitalization, and death from medical causes.

**Methods:**

A cohort of 18,215 patients with substance-related disorders (SRD) recruited in addiction treatment centers was investigated using Quebec (Canada) health administrative databases. A latent class analysis was produced, identifying three profiles of quality of outpatient care use, while multinomial and logistic regressions tested associations with patient characteristics and adverse outcomes, respectively.

**Results:**

Profile 1 patients (47% of the sample), labeled “Low outpatient service users”, received low quality of care. They were mainly younger, materially and socially deprived men, some with a criminal history. They had more recent SRD, mainly polysubstance, and less mental disorders (MD) and chronic physical illnesses than other Profiles. Profile 2 patients (36%), labeled “Moderate outpatient service users”, received high continuity and intensity of care by general practitioners (GP), while the diversity and regularity in their overall quality of outpatient service was moderate. Compared with Profile 1, they  were older, less likely to be unemployed or to live in semi-urban areas, and most had common MD and chronic physical illnesses. Profile 3 patients (17%), labeled “High outpatient service users”, received more intensive psychiatric care and higher quality of outpatient care than other Profiles. Most Profile 3 patients lived alone or were single parents, and fewer lived in rural areas or had a history of homelessness, versus Profile 1 patients. They were strongly affected by MD, mostly serious MD and personality disorders. Compared with Profile 1, Profile 3 had more frequent ED use and hospitalizations, followed by Profile 2. No differences in death rates emerged among the profiles.

**Conclusions:**

Frequent ED use and hospitalization were strongly related to patient clinical and sociodemographic profiles, and the quality of outpatient services received to the severity of their conditions. Outreach strategies more responsive to patient needs may include motivational interventions and prevention of risky behaviors for Profile 1 patients, collaborative GP-psychiatrist care for Profile 2 patients, and GP care and intensive specialized treatment for Profile 3 patients.

**Supplementary Information:**

The online version contains supplementary material available at 10.1186/s13011-022-00511-0.

## Background

Patients with substance-related disorders (SRD), including substance-induced or substance use disorders, substance intoxication or withdrawal, are known to use acute care frequently [1, 2]. A 2014–18 US study reported that 9.4% of annual emergency department (ED) use and 11.9% of hospitalizations were related to substance use disorders [3]. ED use by patients with SRD may be related to intoxication, overdose, withdrawal, or associated health conditions [4]. Frequent ED use and hospitalizations are costly, and are key indicators of adverse outcomes [5], often indicating lack of appropriate outpatient care as well [6]. More intensive and continuous outpatient care by various health professionals is usually required for patients affected by multimorbidity [7]. Patients with SRD are often affected by co-occurring chronic physical illnesses and mental disorders (MD), increasing the risk of acute care use [8] and premature death [9]. SRD also increases the risk of death by accident, suicide, or homicide [10, 11]. Yet, high quality of care, defined as higher SRD treatment frequency [8] and overall continuity of care [12, 13], reportedly decrease ED use and hospitalizations among patients with SRD. SRD treatment completion is also linked to improved health conditions and lower risk of death [14]. Strengthening the quality of outpatient care and treatment adherence in response to the needs of patients with SRD and co-occurring disorders are thus key issues. Identifying profiles of patients with SRD based on the quality of outpatient care received may support the formulation of strategies adapted to each specific profile, to reduce risks of frequent ED use, hospitalizations, and death.

Few studies have elaborated profiles of outpatient service use among patients with SRD [15–19]. These studies mostly assessed the types of services patients used, comparing in hospital settings profiles of patients with higher use of SRD, MD or other programs [15, 17], or main SRD treatment (e.g., Alcoholics Anonymous) [16], principal clinicians consulted (e.g., psychiatrist, psychologist) [16, 18, 19], or outpatient versus acute care used [15, 18]. Most studies included patients with SRD in general [15, 17, 18], whereas a few focused on MD-SRD [19] or alcohol-related disorders only [16]. Some studies investigated the evolution of service use profiles according to age [19] over a three- to eight-year period, leading to treatment disengagement [17] or reduced alcohol use [16]. Profiles related to low [15, 18, 19] and multiple [16, 19] service users have been identified, as well as profiles of patients using mainly Alcoholics Anonymous [16], psychiatric services [15, 17–19] or general practitioners (GP) [19]. Most studies have linked patient service use profiles to their sociodemographic and clinical characteristics, with MD as the main associated clinical variable tested. Most profiles of low service users have consisted of men and younger patients [18], whereas profiles showing more GP use included more women and older patients [19]. More multiple service users had several SRD or MD [19]. Profiles of patients who used mainly psychiatric services included more patients with co-occurring SRD and serious MD [17].

Previous typologies have rarely integrated quality of care indicators and considered a limited number of services used by patients with SRD in only one or few settings. Patients using addiction treatment centers or specialized SRD care, who often present complex health issues and poor social conditions [18], may experience reduced adverse outcomes if given intensive diversified services along with high regularity and continuity of care. As well, patients with SRD often require multiple episodes of care as treatment adherence is a key issue for them, affecting their recovery. Yet no typology to date has linked profiles of outpatient service use with acute care use and risk of death. Identifying such profiles may help improve services and patient conditions, especially if associated with sociodemographic and clinical patient characteristics, which also remain insufficiently investigated. This study thus seeks to identify outpatient service use profiles among patients with SRD recruited in addiction treatment centers, based on quality-of-care indicators, and linked the profiles with sociodemographic and clinical characteristics, and the adverse outcomes associated with frequent ED use, hospitalization, or death.

## Methods

### Study context

The province of Quebec (Canada) has a public healthcare system. Specialized public SRD services are provided by addiction treatment centers, treating roughly 10% of the most vulnerable SRD populations [20]. These centers offer SRD programs like detoxification, opioid agonist treatment and rehabilitation, and include brief intervention units accessible through self-referral, referral from primary care services, or court order. They complement primary care services, including care provided by GP, most of whom work in family medicine groups, or psychosocial teams operating in community healthcare centers. Family medicine groups integrating clinicians like nurses and social workers require patient registration and provide extended medical coverage to ensure better continuity of care.

### Study sample, sources, and design

**Fig. 1:**
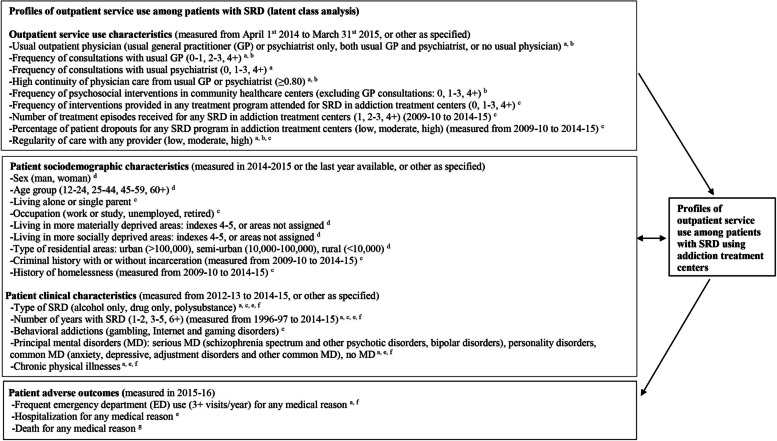
Analytical framework: Profiles of outpatient service use, associated sociodemographic and clinical characteristics, and adverse outcomes among patients with substance-related disorders (SRD) using addiction treatment centers. ^a^ *Régie de l’assurance maladie du Québec* (RAMQ, Physician Claims database); ^b^ *Banque de données communes des urgences* (BDCU, ED database); ^c^ *Maintenance et exploitation des données pour l’étude de la clientèle hospitalière* (MED-ECHO, Hospital Inpatient and Day Surgery database); ^d^ *Système d’information clientèle pour les services de réadaptation en dépendances* (SIC-SRD, Addiction Treatment Center database, including SRD and behavioral addictions based on standardized instruments); ^e^ *Système d’information permettant la gestion de l’information clinique et administrative dans le domaine de la santé et des services sociaux* (I-CLSC, Psychosocial Interventions in Community Healthcare Centers, including GP working on salary); ^f^ *Fichier d’inscription des personnes assurées* (FIPA, Health Insurance Registry); ^g^ *Fichier des décès du Registre des évènements démographiques* (RED, Vital Statistics Death database). For definitions of the variables included in the study, see footnotes in Table 1 or the Methods section. Details on diagnostic codes are presented in Appendix 1

Data were taken from a cohort of 18,697 patients with SRD who used one of 14 Quebec addiction treatment centers (of 16, in total) from April 1, 2012 to March 31, 2013. Administrative data on these patients had to be available in the databases of these centers (SIC-SRD) for the financial years 2009–10 to 2015–16. Patients also had to be Quebec residents, 12+ years old, and eligible for the Quebec Health Insurance Plan (*Régie de l’assurance maladie du Québec*, RAMQ) between 1996–97 and 2015–16. Patients were excluded if they were hospitalized > 90 days in 2014–15, which would have hindered the assessment of outpatient quality of care, or if they died between 2012–13 and 2014–15. Data from addiction treatment centers included patient sociodemographic characteristics, type of SRD, and services received in these centers. The RAMQ keeps billing data for most physician services, excluding 6% occurring outside the public system [21] and integrates various sub-databases: e.g., on ED use, hospitalization, psychosocial interventions in community healthcare centers, and death records. The data from all databases were merged for each patient and each year using a unique RAMQ identifier matched with the SIC-SRD database. Figure 1, the analytical framework for the study, identifies databases linked to each study variable, including the timeframe for their measurement. RAMQ diagnostic codes were framed by the International Classification of Diseases, Ninth and Tenth Revisions (Appendix 1). The SIC-SRD integrated standardized instruments which measured the presence of SRD (yes/no), based on the Addiction Severity Index [22, 23] or the Global Appraisal of Individual Needs [24].

Profiles of patients with SRD were created based on their outpatient service use in 2014–15, except for percentages of dropouts from any SRD programs in addiction treatment centers measured from 2009–10 to 2014–15. Patient sociodemographic characteristics were measured in 2014–15 or in the last year for which data were available, excluding criminal history or history of homelessness, which were measured from 2009–10 to 2014–15. Patient clinical characteristics were measured from 2012–13 to 2014–15, except for number of years with SRD, which was measured from 1996–97 to 2014–15. Adverse outcomes were measured in 2015–16. The Quebec Commission for Access to Information granted access to the databases without requiring informed consent from patients. The ethics review board of a health and social service organization approved the study protocol.

### Study variables

Outpatient service use characteristics included: having a usual outpatient physician and frequency of patient consultations with this physician; high continuity of physician care; frequency of psychosocial interventions received in community healthcare centers or from any SRD service in addiction treatment centers; percentage of patient dropouts from any SRD programs in addiction treatment centers; and regularity of outpatient care. “Usual GP”, a proxy for family doctor, was defined as having at least two consultations with the same GP, or with at least two GP working in the same family medicine group. “Usual psychiatrist” was also defined by a minimum of two consultations, or only one if the patient had also consulted a usual GP, which was considered a proxy for collaborative care [25]. Minimal acceptable intensity of care was defined as receiving 4+ consultations or interventions/year [26–28]. Continuity of physician care was measured with the Usual Provider Continuity Index, which describes the proportion of consultations with the usual GP or psychiatrist, of all GP and psychiatrists consulted in outpatient care, including in walk-in clinics; a score of ≥0.80 is considered high continuity of care [29]. SRD treatment dropout referred to any episode of SRD treatment, as registered in the databases of addiction treatment centers. Regularity of outpatient care was measured considering all outpatient providers in the study and expressed as the number of 3-month periods during which patients used at least one outpatient service. Outpatient service use thus included five quality of care measurements: diversity (biopsychosocial services), intensity, continuity and regularity of service use, and adhesion to SRD services.

Sociodemographic characteristics included: sex, age group, living alone or as a single parent, occupation (work or study, unemployed, retired), living in more materially and socially deprived areas, type of residential area (e.g., urban), criminal history, and history of homelessness. Based on the smallest dissemination areas corresponding to zip codes used in the 2011 Canadian census, the Material Deprivation Index integrated ratios for population employment, average income, and education levels lower than high school. The Social Deprivation Index was used to estimate the proportion of patients living alone, patients with single civil status, and single-parent families [30]. Data taken from both indexes were classified in quintiles, then regrouped as less deprived (1–2-3) or more deprived areas (4–5, or not assigned: e.g., nursing home residents, homeless individuals).

Clinical characteristics included: type of SRD, number of years with SRD, principal MD, and chronic physical illnesses. Type of SRD included exclusive groups of disorders related to alcohol only, drugs only, and polysubstance use. Also referring to exclusive groups, principal MD included serious MD (schizophrenia spectrum and other psychotic disorders, bipolar disorders), personality disorders, and common MD (e.g., anxiety, depressive and adjustment disorders), in that order.

Adverse outcomes included frequent ED use and hospitalizations for any medical reason, and death from any medical cause. Frequent ED use was defined as 3+ visits/year, a standard designation for this variable [7, 31].

### Data analysis

Latent class analysis (LCA) [32, 33] was used to identify patient profiles based on quality of outpatient service use. Compared to standard cluster analysis with an arbitrarily chosen distance measure, LCA allows for statistical testing of model fit with membership probabilities computed from the estimated model parameters [34]. The optimal number of latent classes was determined during the initial step of the analyses, where a serie of increasingly complex models (adding classes) was estimated. In relation to the pertinence of clinical results observed, Akaike Information Criteria (AIC) [35], Bayesian Information Criteria (BIC) [36] and the entropy value [37] were used for selecting the final analytical classification model. Associations between latent class memberships, patient sociodemographic and clinical characteristics were then tested using bivariate analyses (chi-squared tests adjusted with the Holm-Bonferroni method) and a multivariate multinomial logistic regression. As a final step, relationships between class memberships and adverse outcomes were tested using logistic regressions, adjusted for age and sex. LCA was performed with SAS 9.4 [38], and other analyses using Stata 17 [39].

## Results

### Sample characteristics

Of the initial 18,697-member cohort, 115 patients hospitalized 91+ days in 2014–15 were excluded, as were 367 who died between 2012-13 and 2014–15. Of 18,215 patients studied, 65% were men, 45% were 25–44 years old, 46% lived alone or were single parents, 54% were unemployed, while 56% lived in more materially deprived areas and 62% in more socially deprived areas (Table 1). Some patients had a criminal history (19%) or a history of homelessness (13%). Nearly half of patients (49%) had polysubstance-related disorders, while 55% had SRD for more than 2 years. Most patients (69%) had MD, 37% of which were mainly common MD and 22% serious MD, while 37% of patients had chronic physical illnesses. Almost half of patients (47%) had no usual physician. A minority of them received intensive care (4+ consultations/year) with their usual GP (23%) or psychiatrist (10%), or with psychosocial clinicians in either community healthcare centers (20%) or in addiction treatment centers (21%). A minority of patients (44%) received high continuity of physician care; 43% had high rates of treatment dropout from addiction treatment centers; and 30% received high regularity of outpatient care. At 12-month follow-up, 18% were found to be frequent ED users, 17% were hospitalized, and 1% had died.

**Table 1:** Characteristics of patients using addiction treatment services (*N* = 18,215, or other as specified)

	*n*	%
**Outpatient service use characteristics** (measured in 2014–15 (April 1–March 31), or other as specified)		
Usual outpatient physician ^a^		
Usual general practitioner (GP) only	6503	35.70
Usual psychiatrist only	1278	7.02
Both GP and psychiatrist	1847	10.14
No usual physician	8587	47.14
Frequency of consultations with usual GP ^a^		
0–1	9865	54.16
2–3	4243	23.29
4+	4107	22.55
Frequency of consultations with usual psychiatrist ^a^		
0	15,090	82.84
1–3	1375	7.55
4+	1750	9.61
High continuity of physician care from usual GP or psychiatrist (≥0.8) ^b^	7945	43.62
Frequency of psychosocial interventions received in community healthcare centers (excluding GP consultations) ^c^		
0	10,905	59.87
1–3	3744	20.55
4+	3566	19.58
Frequency of interventions provided in any treatment programs attended for SRD in addiction treatment centers ^d^		
0	12,564	68.98
1–3	1756	9.64
4+	3895	21.38
Percentage of patient dropouts from any SRD program in addiction treatment centers (measured from 2009 to 10 to 2014–15) ^e^		
Low (0 to 33%)	6511	35.74
Moderate (34 to 66%)	3856	21.17
High (67 to 100%)	7848	43.09
Regularity of outpatient care with any provider (3 months per period) ^f^		
Low (services received in 1 or 2 periods, or less than 2 services received)	9449	51.87
Moderate (services received in 3 periods)	3373	18.52
High (services received in 4 periods)	5393	29.61
**Patient sociodemographic characteristics** (measured in 2014–15 or the last year available, or other as specified)		
Men	11,929	65.49
Age group (years)		
12–24	3790	20.81
25–44	8183	44.92
45–59	4889	26.84
60+	1353	7.43
Living alone or single parent (*n* = 16,381)	7518	45.89
Occupation		
Work or study	8096	44.44
Unemployed	9923	54.48
Retired	196	1.08
Living in more materially deprived areas: Indexes 4–5 or areas not assigned ^g^	10,205	56.03
Living in more socially deprived areas: Indexes 4–5 or areas not assigned ^g^	11,278	61.92
Type of residential areas ( = 18,197)		
Urban areas (> 100,000)	9417	51.75
Semi-urban areas (10,000 to 100,000)	5323	29.25
Rural areas (< 10,000)	3457	19.00
Criminal history with or without incarceration (measured from 2009 to 10 to 2014–15)	3476	19.08
History of homelessness (measured from 2009 to 10 to 2014–15)	2446	13.43
**Patient clinical characteristics** (measured from 2012 to 13 to 2014–15, or other as specified)		
Type of substance-related disorders (SRD)		
Drugs only	5440	29.86
Alcohol only	3768	20.69
Polysubstances	9007	49.45
Number of years with SRD (measured from 1996 to 97 to 2014–15)		
1–2	8244	45.26
3–5	5611	30.80
6+	4360	23.94
Principal mental disorders (MD) ^h^		
Serious MD	3930	21.58
Personality disorders	1971	10.82
Common MD	6682	36.68
No MD	5632	30.92
Chronic physical illnesses ^i^	6771	37.17
**Patient adverse outcomes** (measured in 2015–16)		
Frequent emergency department (ED) use (3+/year) for any medical reason ^j^	3272	17.96
Hospitalizations for any medical reason	3095	16.99
Death from any medical cause	164	0.90

^a^Usual outpatient physician includes general practitioner (GP) and psychiatrist. Usual GP is a proxy for “patient family physician”, as this information is not available in administrative databases. Usual GP is one with whom the patient had at least two consultations or at least two consultations with GP working in the same family medicine group, as defined in the Methods section. Usual psychiatrist is defined as one that followed the patient in outpatient care at least twice. Alternatively, patients who made only one outpatient consultation with a psychiatrist had to have consulted their GP at least twice, which was considered a proxy for collaborative care (see references in Methods section)

^b^Continuity of physician care is measured with the Usual Provider Continuity Index, describing the proportion of consultations with the usual GP or psychiatrist of all GP and psychiatrists consulted in outpatient care, including consultations in walk-in clinics. A score ≥ 0.80 is considered high continuity of care. References are provided in Methods section

^c^Community healthcare centers provide mainly psychosocial interventions delivered through multidisciplinary teams (e.g., social workers, nurses, psychologists). These services are thus complementary to the care provided by GP, and both are primary care (or first line) services

^d^Treatment programs offered in addiction treatment centers included: medical activities (e.g., opioid agonist treatment), specialized addiction services, either internal (e.g., detoxification treatment) or external (e.g., counseling, rehabilitation), and brief treatment (see Methods section)

^e^The addiction treatment center database (SIC-SRD) provided reasons justifying patient case closure (e.g., treatment dropout, treatment completion, patient relocation to another area not covered by the center). It was possible to calculate the percentage of dropouts per patient, accounting for all programs used by the patient over the 6-year data collection period

^f^Outpatient care integrates care from GP, psychiatrists, and clinicians from community healthcare centers and addiction treatment centers. This variable measured how care, whether regulatory or not, was provided during the 12-month period. Patients could receive high regularity of care (interventions received one or several times every 4 months within the 12-month period), moderate regularity of care (interventions received for 3 periods of 3 months in the 12-month period) or low regularity of care (all other possibilities)

^g^Material and social deprivation indexes are related to the smallest residential dissemination areas (zip code areas), based on the 2011 Canadian census. For this study, quintiles were regrouped into two levels representing the less (1–3) and more (4–5 or not assigned) deprived areas. “Not assigned” areas related to missing address or living in an area where index assignment was not feasible. An index cannot usually be assigned to residents of nursing homes or to homeless individuals. The “not assigned areas” were integrated with indexes 4–5 as the more socially deprived areas, as they usually related also to deprived populations

^h^Principal MD include inclusive and hierarchical groups representing the most serious MD. For example, if a patient had both bipolar disorders and personality disorders, then he/she was classified with bipolar disorders, and integrated within serious MD

^i^Chronic physical illnesses included: renal failure, cerebrovascular illnesses, neurological illnesses, endocrine illnesses, tumor without or with metastasis, chronic pulmonary illnesses, diabetes complicated and uncomplicated, cardiovascular illnesses, and other chronic illness categories (e.g., blood loss anemia) (see Appendix 1)

^j^A minimum of three visits per year is the standard definition for frequent ED use, based on previous research. References are provided in the methods section

### Patient profiles

A three-class model was selected as the final analytical classification model, based on the largest entropy value (0.99) and smaller AIC and BIC criteria. Accounting for 47% of the sample, Profile 1 was labeled “Low outpatient service users”. Profile 1 included only patients without a usual physician and without continuity of physician care. A few of these patients received psychosocial interventions either from community healthcare centers (30%) or addiction treatment centers (23%), with only 12–15% receiving more intensive care (4+ interventions/year). Profile 1 had the highest dropout rate from addiction treatment centers (46%) and included the smallest percentage of patients receiving high regularity of outpatient care (9%).

Representing 36% of the sample, Profile 2 was labeled “Moderate outpatient service users”. Profile 2 included patients with at least two consultations with their usual GP, with 48% receiving more intensive GP care (Table 2). Profile 2 featured the most patients with high continuity of physician care (84%), although none had a usual psychiatrist. This profile had the second highest number of patients receiving psychosocial interventions in community healthcare centers (44%) and SRD treatments in addiction treatment centers (35%), with one quarter of them receiving more intensive care. Most patients were provided with high (38%) or moderate (31%) regularity of outpatient care.

**Table 2:** Characteristics of the 3-class model based on outpatient care use characteristics (*N* = 18,215)

	Profile 1 Low outpatient service users	Profile 2 Moderate outpatient service users	Profile 3 High outpatient service users
	%	%	%
**Group size**	**47.14**	**35.70**	**17.16**
**Outpatient service use characteristics** (measured in 2014–15 (April 1–March 31), or other as specified)			
Usual outpatient physician ^a^			
Usual general practitioner (GP) only	0.00	100.00	0.00
Usual psychiatrist only	0.00	0.00	40.90
Both GP and psychiatrist	0.00	0.00	59.10
No usual physician	100.00	0.00	0.00
Frequency of consultations with usual GP ^a^			
0–1	100.00	0.00	40.90
2–3	0.00	51.99	27.58
4+	0.00	48.01	31.52
Frequency of consultations with usual psychiatrist ^a^			
0	100.00	100.00	0.00
1–3	0.00	0.00	44.00
4+	0.00	0.00	56.00
High continuity of physician care from usual GP or psychiatrist (≥0.8) ^b^	0.00	83.87	79.71
Frequency of psychosocial interventions received in community healthcare centers (excluding GP consultations) ^c^			
0	69.88	55.90	40.61
1–3	18.06	22.59	23.17
4+	12.06	21.51	36.22
Frequency of interventions provided in any treatment programs attended for SRD in addiction treatment centers ^d^			
0	76.67	65.08	55.94
1–3	8.34	10.76	10.88
4+	14.99	24.16	33.18
Percentage of patient dropouts from any SRD program in addiction treatment centers (from 2009 to 10 to 2014–15) ^e^			
Low (0 to 33%)	34.76	37.41	34.98
Median (34 to 66%)	19.05	21.92	25.44
High (67 to 100%)	46.19	40.67	39.58
Regularity of outpatient care with any provider (3 months per period) ^f^			
Low (services received in 1 or 2 periods or less than 2 services received)	82.83	31.28	9.70
Median (services received in 3 periods)	8.59	30.68	20.48
High (services received in 4 periods)	8.58	38.04	69.82

^a^See note ^a^ below Table 1

^b^See note ^b^ below Table 1

^c^See note ^c^ below Table 1

^d^See note ^d^ below Table 1

^e^See note ^e^ below Table 1

^f^See note ^f^ below Table 1

Representing 17% of the sample, Profile 3 was labeled “High outpatient service users” and included patients with both a usual GP and psychiatrist (59%) or a usual psychiatrist only (41%). This profile had the most patients (56%) who received intensive psychiatric care and the second highest rating (80%) for high continuity of physician care. These patients also received the highest percentage (59%) of follow-up by community healthcare centers (59%) and addiction treatment centers (44%), with one third receiving intensive care in these centers. This profile featured the highest patient percentage for regularity of outpatient care (70%).

### Patient sociodemographic and clinical characteristics associated with profiles

Compared to Profile 1 (Low outpatient service users: reference group), Profiles 2 (Moderate outpatient service users) and 3 (High outpatient service users) included more women, more patients having SRD for 3+ years as well as serious MD, personality disorders, common MD, and chronic physical illnesses. More particularly, Profile 3 included patients with 53-, 24- and 9-times higher risk of serious MD, personality disorders, and common MD, respectively, than those in Profile 1. Compared with Profile 1 patients, fewer of those in Profiles 2 and 3 lived in more materially and socially deprived areas or had a criminal history. Profile 2 patients were more likely to be 25+ years old and less likely to be either unemployed, living in semi-urban areas or affected by polysubstance-related disorders compared with Profile 1 patients. Those in Profile 3 were more likely to live alone or to be single parents and were less likely to be 45+ years old, to live in rural areas, or to have a history of homelessness compared with their Profile 1 counterparts (Table 3).

**Table 3:** Comparisons between service use profiles and sociodemographic and clinical correlates (*N* = 18,125)

	Profile 1 Low outpatient service users		Profile 2 Moderate outpatient service users		Profile 3 High outpatient service users		Bivariate analysis: chi-square test
	%	RR	%	RR	%	RR	***P***-value
**Group size**	**47.14**		**35.70**		**17.16**		
**Patient sociodemographic correlates** (measured in 2014–15 or the last year available, or other as specified)							
Women (ref.: men)	26.23	–	42.96	2.05*	39.68	1.72*	< 0.05
Age group (years) (ref.: 12–24 years)							< 0.05
12–24	27.02	–	14.51	–	16.86	–	
25–44	44.99	–	42.13	1.48*	50.56	0.94	
45–59	22.38	–	32.72	1.86*	26.85	0.76*	
60+	5.61	–	10.64	2.16*	5.73	0.57*	
Living alone or single parent (ref.: none)	41.09	–	49.60	1.07	51.11	1.12*	< 0.05
Occupation (ref.: work or study)							< 0.05
Work or study	49.50	–	43.95	–	31.58	–	
Unemployed	49.67	–	54.71	0.83*	67.20	1.12	
Retired	0.83	–	1.34	0.83	1.22	1.50	
Living in more materially deprived areas: Indexes 4–5 or areas not assigned ^a^ (ref.: indexes 1–3)	57.25	–	54.39	0.87*	56.06	0.81*	
Living in more socially deprived areas: Indexes 4–5 or areas not assigned ^a^ (ref.: indexes 1–3)	61.28	–	61.16	0.84*	65.25	0.85*	
Type of residential areas (ref.: urban areas)							< 0.05
Urban areas (> 100,000)	49.45	–	53.39	–	54.66	–	
Semi-urban areas (10,000 to 100,000)	30.52	–	27.48	0.89*	29.45	0.99	
Rural areas (< 10,000)	20.03	–	19.13	0.90	15.89	0.78*	
Criminal history with or without incarceration (measured from 2009 to 10 to 2014–15) (ref.: none)	21.93	–	16.39	0.83*	16.86	0.71*	< 0.05
History of homelessness (measured from 2009 to 10 to 2014–15) (ref.: none)	12.10	–	13.59	0.98	16.74	0.79*	< 0.05
**Patient clinical correlates** (measured 2012–13 to 2014–15 or other as specified)							
Type of substance-related disorders (SRD) (ref.: drug only)							< 0.05
Drugs only	33.53	–	27.61	–	24.51	–	
Alcohol only	19.33	–	25.03	0.95	15.36	0.98	
Polysubstances	47.14	–	47.36	0.91*	60.13	0.99	
Number of years with SRD (measured from 1996 to 97 to 2014–15) (ref.: 1–2 years)							< 0.05
1–2	56.55	–	40.58	–	23.97	–	
3–5	26.33	–	33.03	1.55*	38.46	2.15*	
6+	17.12	–	26.39	1.66*	37.57	2.43*	
Principal mental disorders (MD) ^b^ (ref.: none)							< 0.05
None	46.44	–	24.02	–	2.62	–	
Serious MD	12.87	–	14.99	1.64*	59.21	53.68*	
Personality disorders	7.70	–	12.12	2.21*	16.70	24.85*	
Common MD	32.99	–	48.87	2.54*	21.47	9.78*	
Chronic physical illnesses ^c^ (ref.: none)	26.09	–	46.69	1.80*	47.84	1.54*	< 0.05

* = *p*-value < 0.05. RR = risk ratio. Each RR is adjusted for the remaining variables in the model

^a^ See note ^g^ below Table 1

^b^ See note ^h^ below Table 1

^c^ See note ^i^ below Table 1

### Patient adverse outcomes associated with the profiles

Controlling for patient age and sex, the odds of being frequent ED users or hospitalized for any medical reason were higher in Profile 3 (High outpatient service users) followed by Profile 2 (Moderate outpatient service users), than in Profile 1 (Low outpatient service users) (Table 4). No significant differences were found among the three profiles regarding death rates from any medical cause.

**Table 4:** Comparisons between service use profiles and adverse outcomes (measured in 2015–16 (April 1–March 31)) (*N* = 18,125)

	Profile 1 Low outpatient service users		Profile 2 Moderate outpatient service users		Profile 3 High outpatient service users	
	%	RR	%	RR	%	RR
**Group size**	**47.14**		**35.70**		**17.16**	
Frequent emergency department (ED) use (3+/year) for any medical reason ^a^	13.38	–	18.95	1.41*	28.51	2.50*
Hospitalizations for any medical reason	11.55	–	18.58	1.50*	28.64	2.90*
Death from any medical cause	0.73	–	1.15	1.26	0.83	1.14

* = *p*-value < 0.05. OR = odds ratio. Each OR is adjusted for age and sex

^a^ See note ^j^ below Table 1

## Discussion

This study demonstrated that only a minority of patients received diversified, intensive, continuous, and regular outpatient follow-up care, even though roughly half of them had complex social and health conditions. More than 40% also experienced high frequencies of SRD treatment dropout. Three profiles regarding quality of outpatient care received were identified among patients with SRD. Profiles 1 and 3 resembled the low [15, 18, 19] and multiple or high service user profiles respectively described in previous studies [16, 19], while results for Profile 2 (Moderate outpatient service users) were similar to findings in previous studies in terms of GP care profiles [19]. This study was original in investigating quality of care issues, as opposed to service use only. It demonstrated that for a great majority of patients with SRD, quality of care needs to be significantly improved and adjusted to their needs.

It was not surprising to find that Profile 1 (Low outpatient service users) was the largest group with 47% of patients, as those with SRD are known to use few outpatient services [40, 41] and to exhibit high dropout rates [42, 43]. Compared with Profiles 2 and 3, Profile 1 patients received very low overall quality of care and had the highest SRD dropout rate, which is easily explained by their sociodemographic and clinical characteristics. Profile 1 included more men and more patients 12–24 years old than other profiles, that is, two groups known to use outpatient services more as a last resort as opposed to women and older patients [15, 18]. Moreover, with more materially and socially deprived patients than those in Profiles 2 and 3, also neighborhoods often associated with criminal activities [44, 45], Profile 1 patients may have faced particularly strong stigma. Previous studies have reported that young people, mainly those affiliated with a “sub-culture of poverty” [46], are more likely to use drugs, deny their SRD, and show reluctance to receive treatment [47]. Moreover, access to SRD treatment [15, 48] and treatment dropout [49, 50] were identified as more associated with younger age groups in previous studies.

Compared with Profiles 2 and 3, Profile 1 also included patients with better health conditions, less chronic or co-occurring MD and chronic physical illnesses, as only a minority had these conditions. These characteristics may explain, in part, why these patients received less outpatient care and had less frequent ED use and hospitalizations than those in other profiles. However, no Profile 1 patient had a usual GP or psychiatrist, and frequent ED use was about twice the rate found in the general Quebec patient population without SRD or MD [51], suggesting that outpatient care needs to be greatly improved. As well, few of these patients received services from either addiction treatment or community healthcare centers, despite being materially and socially deprived patients with SRD for the most part. As patient conditions may rapidly deteriorate with age and chronic SRD [52], prevention and outreach strategies may need particular reinforcement for Profile 1, especially for men and younger patients, who are more reluctant to use outpatient services. In acute care settings, screening, brief intervention, and treatment referral (SBIRT) [53] and motivational interventions might also be deployed to increase access to and continuity of patient care.

As the second largest group (36%), Profile 2 (Moderate outpatient service users) showed more frequent ED use and hospitalizations than Profile 1. However, of all the profiles, Profile 2 patients received the highest continuity and intensity of GP care. The fact that most of these patients had more “chronic” SRD, nearly half SRD co-occurring with chronic physical illnesses or common MD, and were 45+ years of age may explain both their frequent ED use and hospitalizations as well as intense GP care. Older patients are more likely than younger ones to be followed by a GP [54], who more likely treat common MD, especially co-occurring with chronic physical health conditions, than serious MD [55]. Compared with Profile 1 patients, the fact that profile 2 patients were both less likely to be unemployed and live in semi-urban areas might be explained by their older age, better social and material conditions, and less criminal history, as well as by the usually higher percentage of GP working in urban areas [56]. Taken together, the conditions affecting Profile 2 patients, along with the moderate quality of outpatient care they received, including no psychiatric care, might explain their high ED use and hospitalizations. Collaborative care between MD-SRD health specialists might be suggested to facilitate better treatment among Profile 2 patients, most of whom were faced with multimorbidity [57, 58].

Accounting for less than one fifth of the study sample, Profile 3 (High outpatient service users) included patients with more complex health and social conditions, most with chronic multimorbidity especially serious MD and personality disorders, which explains their intensive psychiatric care, more intensive treatment for SRD, and high regularity of care. Profile 3 also had the highest percentage of patients with frequent ED use and hospitalizations, roughly twice the percentage found in Profile 1. Chronic SRD [8], serious MD [59, 60] and personality disorders [61] have been found to increase the risk of frequent ED use and hospitalizations. A majority of patients in Profile 3 also lived alone or were single parents, conditions usually related to more adverse outcomes [62, 63]. The lower proportion of patients living in rural areas found in Profile 3 compared with Profile 1 may be explained by the fact that patients with complex health conditions tend to live in larger cities close to specialized care facilities. All Profile 3 patients had a usual psychiatrist. The intensive psychiatric care received by Profile 3 patients may have also explained why they had less history of homelessness than Profile 1 patients. More Profile 3 patients may have been referred to supervised housing with support, as they tended to be older and to have more chronic morbidity – conditions favoring integration into programs like Housing First [64, 65]. Finally, more than 40% of Profile 3 patients did not receive GP follow-up care, and only a third received intensive SRD treatment, which argues for improving outpatient care even for this patient profile. More GP care and intensive psychosocial interventions including SRD treatments [66], and integration into programs like assertive community treatment [67] or intensive case management [68] could thus be recommended for Profile 3 patients. Further implementation of integrated SRD-MD treatment [69] may also be recommended for these patients, as treatment coordination for MD and SRD in Quebec is insufficient [70].

This study has some limitations. First, administrative health databases were primarily developed for financial purposes, not research. These data are thus only proxy measures for patient needs. Second, some variables that may have impacted profiles of outpatient quality of care or adverse outcomes were not available for this study, including the race or ethnicity of patients as well as the receipt of hospital-based psychosocial care services, and private sector services from psychologists and groups like Alcohol Anonymous, Narcotic Anonymous or harm reduction resources. Finally, the findings may not be generalizable to other contexts, especially healthcare systems or populations with no public insurance or limited access to specialized SRD services.

## Conclusion

Findings from this study demonstrated that high ED use and hospitalizations were strongly related to clinical and sociodemographic characteristics of patients, and that the quality of outpatient services received was proportional to the complexity and severity of their health and social conditions. The percentage of patient deaths did not differ between profiles, probably due to the insufficient sample size within the cohort. Unfortunately, the study found that the overall quality of care for patients with SRD needs to be greatly improved. Only Profile 3 patients received relatively higher quality of care, and these represented one fifth only of all the study patients. About half of the study patients (Profile 1) received almost no service at all. From two thirds (Profile 1) to four out of ten (Profile 3) of the study patients received SRD treatment for the last 12-month period of the study follow-up, and overall dropout from treatment was very high in the cohort. Based on the study results, outreach strategies might include motivational interventions, and prevention of risky behaviors for Profile 1 patients, collaborative GP-psychiatrist care for Profile 2 patients, and GP care and intensive specialized treatment for Profile 3 patients with a view toward better responding to the needs of these patients.

## Supplementary Information

**Additional file 1: Appendix 1.** Codes for substance-related disorders, mental disorders, chronic physical illnesses, and death according to the International Classification of Diseases, Ninth and Tenth revisions.

## Data Availability

In accordance with the applicable ethics regulations in the province of Quebec, the principal investigator is responsible for keeping data confidential.

## Abbreviations

AIC: Akaike Information Criteria
BIC: Bayesian Information Criteria
ED: Emergency departments
GP: General practitioner
LCA: Latent class analysis
MD: Mental disorders
RAMQ: Régie de l’Assurance maladie du Québec
SBIRT: Screening, brief intervention, and treatment referral
SIC-SRD: Addiction Treatment Center Database (Système d’information sur la clientèle des services de réadaptation en dépendances)
SRD: Substance-related disorders
