# Supplementary material for: Profiles of quality of outpatient care use, associated sociodemographic and clinical characteristics, and adverse outcomes among patients with substance-related disorders
Source: Subst Abuse Treat Prev Policy. 2023 Jan 14;18:5. doi: 10.1186/s13011-022-00511-0 (PMC9840840; doi:10.1186/s13011-022-00511-0)
Supplement: Supplementary file 1 — Additional file 1: Appendix 1. Codes for substance-related disorders, mental disorders, chronic physical illnesses, and death according to the International Classification of Diseases, Ninth and Tenth revisions. [file 13011_2022_511_MOESM1_ESM.docx]

**Appendix 1: Codes for substance-related disorders, mental disorders, chronic physical illnesses, and death according to the International Classification of Diseases, Ninth and Tenth revisions**

| **Diagnoses** | ***International Classification of Diseases, Ninth Revision (ICD-9)*** | ***International Classification of Diseases, Tenth Revision, Canada***  **(*ICD-10-CA)*** |
| --- | --- | --- |
| ***Substance-related disorders (SRD)***^a^ | | |
| Alcohol-related disorders | 3030*, 3039*, 3050* (alcohol abuse or dependence); 2910*, 2918* (alcohol withdrawal), 2911*-2915*, 2919*, 3575, 4255, 5353, 5710-5713 (alcohol-induced disorders); 9800, 9801, 9808, 9809 (alcohol intoxication) | F101*, F102* (alcohol abuse or dependence); F103, F104* (alcohol withdrawal); F105-F109, K700*-K704*, K709*, G621*, I426, K292*, K852, K860, E244, G312, G721, O354 (alcohol-induced disorders); F100*, T510, T511*, T518, T519 (alcohol intoxication) |
| Drug-related disorders | 3040-3043, 3044-3049, 3052-3057, 3059 (drug abuse or dependence); 292.0 (drug withdrawal); 2921, 2922, 2928, 2929 (drug-induced disorders); 9650, 9658, 9670, 9676, 9678, 9679, 9694-9699, 9708, 9820, 9828 (drug intoxication) | F111, Fi21, F131, F141, F151, F161, F181, F191, F112, F122 F132, F142, F152, F162, F182, F192 (drug abuse or dependence); F113-F114, F123-F129, F133-F134, F143-F144, F153-F154, F163-F164, F183-F184, F193-F194 (drug withdrawal) F115-F119, F135-F139, F145-F149, F155-F159, F165-F169, F185-F189, F195-F199 (drug-induced disorders); F110, F120, F130, F140, F150, F160, F180, F190, T400-T406, T407, T408, T409, T423, T424, T426, T427, T435, T436, T438, T439, T509, T528, T529 (drug intoxication) |
| ***Mental disorders (MD)***^a^ | | |
| ***Serious MD*** | | |
| Schizophrenia spectrum and other psychotic disorders | 295* (schizophrenic disorders); 297* (paranoid states); 298* (other nonorganic psychoses) | F20* (schizophrenic disorders); F22* (persistent delusional disorders); F23 (acute and transient psychotic disorders); F24* (induced delusional disorder); F25* (schizoaffective disorders); F28* (other psychotic disorder not due to a substance or known physiological condition); F29* (unspecified psychosis not due to a substance or known physiological condition); F448 (other dissociative and conversion disorders); F481 (depersonalization-derealization syndrome) |
| Bipolar disorders | 2960-2966 (manic disorders); 2968 (other affective psychoses); 2969 (unspecified affective psychoses) | F300-F302, F308, F309 (manic episode); F310-F317, F318, 319 (bipolar episode) |
| ***Personality disorders*** | 3010 (paranoid personality disorder); 3011 (affective personality disorder); 3012 (schizoid disorder); 3013, 3014 (obsessive-compulsive personality disorder); 3015 (histrionic personality disorder); 3016 (dependent personality disorder); 3017 (antisocial personality disorder); 3018 (other personality disorders); 3019 (unspecified personality disorder) | F600 (paranoid personality disorder); F61 (mixed and other personality disorders); F340 (cyclothymic disorder); F341 (dysthymic disorder); F601 (schizoid personality); F603 (borderline personality disorder); F605 (obsessive-compulsive personality disorder); F604 (histrionic personality disorder); F607 (dependent personality disorder); F602 (antisocial personality disorder); F609 (unspecified personality disorder); F21 (schizotypal personality); F606 (avoidant personality disorder); F608 (other specified personality disorders); F681 (factitious disorder); F688 (other specified disorders of adult personality and behaviour); F69 (unspecified disorder of adult personality and behaviour) |
| ***Common MD*** | | |
| Depressive disorders | 3004 (neurotic depression) *; 311, 3119* (depressive disorder, not elsewhere classified) | F320- F323 (major depressive disorder, single episode); F328 (other depressive episodes); F329 (depressive episode, unspecified); F330-F334 (major depressive disorder, recurrent); F338 (other recurrent depressive disorders); F339 (recurrent depressive disorder, unspecified); F348 (other persistent mood [affective] disorders); F380, F381 (persistent mood [affective] disorder, unspecified); F388 (other specified mood [affective] disorders); F39 (unspecified mood [affective] disorders); F412* (mixed anxiety and depressive disorder)* |
| Anxiety disorders | 300 (except 3004); 3000 (anxiety states); 3002 (phobic anxiety disorders); 3003 (obsessive-compulsive disorder); 3001 (hysteria); 3006 (other anxiety disorder); 313 (disturbance of emotions specific to childhood and adolescence) | F40 (phobic anxiety disorders); F41(other anxiety disorders); F42 (obsessive-compulsive disorder); F45 (somatoform disorders); F48 (other neurotic disorders); F93, F94 (disturbance of emotions specific to childhood and adolescence) |
| Adjustment disorders | 3090 (brief depressive reaction); 3092 (adjustment reaction with predominant disturbance of other emotions, include: abnormal separation anxiety); 3093 (adjustment reaction with predominant disturbance of conduct); 3094 (adjustment reaction with predominant disturbance of other emotions and conduct); 3098 (other specified adjustment reactions); 3099 (unspecified adjustment reaction) | F430 (acute stress reaction); F431 (post-traumatic stress disorder); F432 (adjustment disorders); F438 (other reactions to severe stress); F439 (reaction to severe stress, unspecified) |
| Other MD | 314 (attention deficit/hyperactivity disorder); 2930, 2931 (transient organic psychotic conditions); 2940, 2941 (other organic psychotic conditions); 2990, 2991*, 2998, 2999 (pervasive developmental disorders); 290, 2941, 3310, 3312 (dementia); 3020-3029 (sexual deviations and disorders); 3070-3079 (special symptoms or syndromes, not elsewhere classified include anorexia nervosa, tics); 312 (disturbance of conduct, not elsewhere classified); 3150-3159 (specific delays in development); 316 (psychic factors associated with diseases classified elsewhere); 317-318 (mental retardation) | F900; F901; F908; F909 (attention deficit/hyperactivity disorder); F060-F069 (other mental disorders due to known physiological condition); F840, F841, F842, F843, F844, F845 (pervasive developmental disorders); F00x-F03, F051, G30, G311(dementia); F500-F502 (eating disorders); F520-F529 (sexual dysfunction, not caused by organic disorder or disease); F510-F515 (nonorganic sleep disorders); F950-F952, F958, F959 (tic disorders); F980-F986, F988, F989 (other behavioral and emotional disorders with onset usually occurring in childhood and adolescence); F630-F633, F638, F639 (habit and impulse disorders); F70-73, F78, F79 (mental retardation) |
| ***Chronic physical illnesses*** ^a, b^ | | |
| Renal failure | 4030, 4031, 4039, 4040, 4041, 4049, 585, 586, 5880, V420, V451, V56 | I120, I131, N18, N19, N250, Z49, Z940, Z992 |
| Cerebrovascular illnesses | 430-438 | G45, G46, I60-I69 |
| Neurological illnesses | 3319, 3320, 3321, 3334, 3335, 3339, 334–335, 3362, 340, 341, 345, 3481, 3483, 7803, 7843 | G10–G12, G13, G20, G21–G22, G254, G255, G312, G318, G319, G32, G35, G36, G37, G40, G41, G931, G934, R470, R56 |
| Endocrine illnesses (hypothyroidism; fluid electrolyte disorders and obesity) | 2409, 243, 244, 2461, 2468; 2536, 276; 2780 | E00, E01, E02, E03, E890; E222, E86, E87; E66 |
| Any tumor with or without metastasis (solid tumor without metastasis; lymphoma) | 140-172, 174, 175, 179-195, 196–199; 200, 201, 202, 2030, 2386, 2733 | C00–C26, C30–C34, C37–C41, C43, C45-C58, C60–C76, C77-C79, C80; C81-C85, C88, C900, C902, C96 |
| Chronic pulmonary illnesses | 490–505, 5064, 5081, 5088 | I278, I279, J40-J47, J60-J64, J65, J66, J67, J684, J701, J703 |
| Diabetes complicated and uncomplicated | 2500-2502, 2503; 2504-2509 | E102-E108, E112-E118, E132-E138, E142-E148; E100, E101, E109, E110, E111, E119, E130, E131, E139, E140, E141, E149 |
| Cardiovascular illnesses (congestive heart failure; cardiac arrhythmias; valvular illnesses; peripheral vascular illnesses; myocardial infarction; hypertension and pulmonary circulation illnesses) | 4021, 4041, 428; 4260, 4267, 4269,4270–4274,4276–4279, 7850, V450, V533; 394–397, 424,7463–7466, V422, V433; 093, 440, 441, 4431– 4439, 4471, 5571, 5579, V434; 4109, 4129; 4010, 4011, 4019, 4020, 4021, 4029, 4050, 405,4051, 4059, 4372; 4150, 4151, 416; 4170, 4178, 4179 | I099, I110, I130, I132, I255, I420, I425–I429, I43, I50, P290; I441–I443, I456, I459, I47–I49, R000, R001, R008, T821, Z450, Z950; A520, I70-I72, I730, I731, I738, I739, I771, I790, K551, K558, K559, Z958, Z959; I05–I08, I091, I098, I34–I39, Q230–Q233, Q238, Q239, Z952, Z953, Z954I210-I214, I219, I220, I221, I228, I229, I252; I101, I100, I11, I1500, I1501, I1510, I1511, I1521, I1581, I1590, I1591, I674; I26, I27, I280, I288, I289 |
| Other chronic physical illness categories (blood loss anemia; ulcer illnesses; liver illnesses; AIDS/HIV; rheumatoid arthritis/collagen vascular illnesses, coagulopathy; weight loss, paralysis; deficiency anemia) | 2800, 2809; 286, 2871, 2873-2875; 5317, 5319, 5327, 5329, 5337, 5339, 5347, 5349; 0702, 0703, 0704, 0705, 4560–4562, 5723, 5728, 5733, 5734, 5739, V427; 042–044; 1361, 446; 7010, 7100–7104, 7105, 7108, 7109, 7112, 714, 7193, 720, 725, 7285, 7288, 7293; 260–263, 7832, 7994; 3341, 342, 343, 3440-3446, 3448, 3449; 2801, 2809, 281, 2859 | D500; K257, K259, K267, K269, K277, K279, K287, K289; B20-B24; D65–D68, D691, D693-D696; B18, I85, I864, I982, K700- K703, K709 K711, K713–K715, K716, K717, K721, K729, K73, K74, K754, K760, K761, K763, K764, K765, K766, K768, K769, Z944; L900, L940, L941, L943, M05, M06, M08, M120, M123, M30, M31, M32–M35, M45, M460, M461, M468, M469; G041, G114, G80, G81, G82, G83; E40–E46, R634, R64, D51–D53, D63, D649; D501, D508; D509 |
| **Cause of death** ^c^ | | |
| Infections | 0-139 | A00-A99, B00-B99, U04 |
| Cancers | 140-239 | C00-C99, D00-D49 |
| Other physical diseases | | |
| Endocrine, nutritional, and metabolic diseases | 240-279 | E00-E99 |
| Diseases of the nervous system | 320-294, 310 | F00-F09, G00-G99, H00-H99 |
| Diseases of the circulatory system | 390-459 | I00-I99 |
| Diseases of the respiratory system | 460-519 | J00-J99 |
| Diseases of the digestive system | 520-579 | K00-K99 |
| Other diseases and symptoms, signs, and abnormal clinical and laboratory findings, not elsewhere classified | All remaining codes not listed, except mental disorders | All remaining codes not listed, excepted mental disorders |
| Injury, poisoning, and certain other consequences of external causes | 800-999 | S00-S99, T00-T99, V00-V99, W00-W99, X00-X99 [except X60-X84], Y00-Y99 [except Y870] |
| Suicide | - | X60-X84, Y870 |

^a^ All diagnoses identified in RAMQ (*Régie de l’assurance maladie du Québec,* Physician Claims database) for the full study period were based on the International Classification of Diseases Ninth Revision (ICD-9), which included a 4-digit code, financial year April 1 to March 31. The Canadian Tenth Revision (ICD-10-CA) was used in MED-ECHO (*Maintenance et exploitation des données pour l’étude de la clientèle hospitalière*, Hospital Inpatient and Day Surgery database) 2006-07+, and in BDCU (*Banque de données communes des urgencies,* emergency department [ED] database). Diagnoses related to all the above databases were considered, and all data integrated each year, for each patient. MED-ECHO is the only database that includes several diagnoses: principal diagnosis and numerous secondary diagnoses. In the databases used in this study, MD were considered only as principal diagnoses, but SRD as both principal and secondary diagnoses, considering that SRD often underdiagnosed. ^b^ The list of chronic physical illnesses is based on an adapted and validated version of the Elixhauser Comorbidity Index, integrating the Charlson Index, which consists of 32 major categories of physical illnesses (see reference in the Methods section). In this list of chronic physical illnesses, three categories of MD and two of SRD (identified with an asterisk [*]) were also included in the list of MD-SRD, thus appearing twice. ^c^ Causes of death were those identified in the *Fichier des décès du Registre des évènements démographiques* (RED, Vital Statistics Death database).
